# The effect of gum chewing on blood GLP-1 concentration in fasted, healthy, non-obese men

**DOI:** 10.1007/s12020-015-0566-1

**Published:** 2015-03-11

**Authors:** Jianping Xu, Xinhua Xiao, Yuxiu Li, Jia Zheng, Wenhui Li, Qian Zhang, Zhixin Wang

**Affiliations:** Department of Endocrinology, Peking Union Medical College Hospital, Peking Union Medical College, Chinese Academy of Medical Sciences, The Ministry of Health Key Laboratory of Endocrinology, Beijing, 100730 China

**Keywords:** Glucagon-like peptide-1 (GLP-1), Chewing, Blood glucose, Insulin, Glucose-dependent insulinotropic peptide (GIP)

## Abstract

We evaluated the effect of chewing on blood GLP-1 concentration by having volunteers to chew sugarless gum. Our intention was to explore the neural mechanisms regulating the secretion of glucagon-like peptide-1(GLP-1). After fasting for 12 h, 12 healthy male, non-obese volunteers (18 < BMI < 30), were asked to chew sugarless gum at a frequency of 80 times every 2 min for a total of 30 min. Blood samples were collected before the start of chewing and 5, 10, 15, 20, 25, and 30 min after the start of chewing. Satiety and hunger were evaluated on a scale from 0 to 100 at each time point. Compared with the control group, the test group’s satiety was increased at 15, 25, and 30 min (*p* = 0.043, *p* = 0.014 and *p* = 0.018, respectively) after they began chewing sugarless gum 80 times every 2 min. The blood GLP-1 level of the test group at 30 min was 49.6 ± 20.3 pmol/l, significantly higher than that of the control group (38.9 ± 20.9 pmol/l; *p* = 0.031). There was no significant difference in the test group’s GLP-1 concentration at each time point. In the control group, compared to baseline, the GLP-1 concentrations at 15, 25, and 30 min were significantly decreased (*p* = 0.042, *p* = 0.0214 and *p* = 0.012, respectively). No significant differences in the blood concentration of glucose, insulin and GIP or hunger were observed between groups. Our study suggests that fasting sugarless gum chewing can increase satiety and reduce the decrease in GLP-1 concentration.

## Introduction

GLP-1 is synthesized in and secreted from enteroendocrine L cells that were found throughout the small and large intestine [1]. The constant basal secretion of GLP-1 from enteroendocrine cells is rapidly augmented by the ingestion of luminal nutrients, including carbohydrates, fats, and proteins [2]. GLP-1 is extremely susceptible to the catalytic activity of the enzyme dipeptidyl peptidase IV (DDP-IV) [3]. Only approximately 10–15 % of newly secreted GLP-1 enters the systemic circulation in its intact form [4]. This insulinotropic activity has been applied to the treatment of type 2 diabetic patients in the form of a new class of antidiabetic agents comprised GLP-1 receptor agonists and dipeptidylpeptidase 4 (DPP-4) inhibitors [5].

Mastication, which serves the physiological function of mechanically breaking food down into small particles suitable for the gastrointestinal absorption of nutrients, influences postprandial plasma glucose concentrations. Compared with typical eating habits, the deliberately thorough mastication of a test meal was reported to be effective in reducing postprandial plasma glucose concentrations in subjects with normal glucose tolerance, most likely because of greater early-phase insulin secretion [6]. If mastication can effect postprandial plasma GLP-1 concentration is not known.

Gum chewing is a voluntary physiological gross motor activity that uses numerous functional neuroanatomical pathways. Gum chewing has been associated with many physiological changes, including increased blood flow in the cerebral and orofacial region, which may account for its association with increased alertness and improved memory [7].

Suggestions that chewing gum may positively influence energy balance and facilitate weight loss have not been convincingly demonstrated. In previous short-term studies, gum chewing has been shown to reduce appetite and food intake [8].

Recently, many scientists have contributed to research examining the effect of gum chewing on weight loss; however, these researchers have reached different conclusions. Hetherington and Regan [9] found that chewing gum for at least 45 min significantly suppressed self-reported hunger, appetite, and snack cravings and promoted satiety. Thus, their study demonstrated the benefits of chewing gum as an aid in appetite control. In 2013, Japanese scholars studied a group of healthy volunteers found that chewed 30 times per bite had GLP-1 concentration that were significantly higher than those of the normal group. One possibility is that chewing 30 times per bite increased the volume of glucose absorption via thorough mastication and the extensive breakdown of carbohydrates [10]. Mattes and Considine [11] found that chewing gum had no effects on appetite sensations or gut peptide concentrations [11].

We suggest that chewing gum can increase GLP-1 secretion and improve satiety.

## Methods

### Subjects

We have received approval from Ethic Committee of Peking Union Medical College Hospital for this study. Participants were recruited via public announcements. Twelve male volunteers provided voluntary consent. Screening prior to the study was conducted to ensure that they met study criteria, i.e., were in good health (not taking medications, no chronic diseases, diabetes or allergies, teeth in a good state of repair). The participants’ height in bare feet was measured. Fasting-state body weight was measured to the nearest 0.1 kg. Eligibility was based on the following criteria: 18–50 years of age; body mass index 18 kg/m^2^ < BMI < 30 kg/m^2^; good health; not initiating or terminating the use of medications reported to affect appetite or body weight during the proposed study period.

### Study protocols

After an overnight fast, the healthy volunteers came to the Endocrinology Department at the Peking Union Medical College Hospital at 8 a.m. Their weight, height, blood pressure, and heart rate were measured, and they were interviewed regarding their past history and health condition. In a within-subject randomized cross-over comparison of hormone concentrations in plasma, 12 subjects were given sugarless gum. On one occasion the gum was chewed (test day), on the other they did not chew the gum (control day).

On each test occasion, after an overnight fast, a catheter was placed into the subject’s antecubital vein and kept patent for half an hour. On the test day, the participants chewed sugarless gum (approximately 1.4 g) for half an hour. The chewing frequency was controlled at 80 times every 2 min. Chewing continued for half an hour. A nurse was responsible for keeping time using a stopwatch. Venous blood was drawn immediately before the volunteers began chewing (0 min) and 5, 10, 15, 25, and 30 min after chewing began. A 2-ml blood sample was drawn in BD TM P800 (Franklin Lakes, NJ, USA) Blood Collection Tubes which was loaded with DPPIV, lipase, and proteinase inhibitor, then spun in a refrigerated centrifuge, and aliquots of plasma were frozen immediately at −80 °C.

Three days later, on the other occasion the individuals returned to the hospital after an overnight fast, and the same tests were performed with the patients chewing nothing, as a control measure.

### Laboratory analysis

We outsourced the testing of blood GLP-1 and GIP concentration to the Beijing North Institute of Biological Technology (Beijing, China). The active GLP-1 [GLP-1-(7-36 amide) and GLP-1-(7-37)] concentration were analyzed using a commercially available RIA assay kit (GLP1A-35HK; Millipore, 6 Research Park Drive, St. Charles, Missouri 63304, USA), and plasma total GIP was analyzed using a commercially available enzyme-linked immunosorbent assay kit (EZRMGIP-55 K; Millipore, Billerica, MA). The intra- and interassay variations for active GLP-1 were 4.8 and 9.7 %, respectively. The intra- and interassay variations for GIP were 5 and 9.6 %, respectively. Glucose was measured using a Roche Accu-Check Performa clinical analyzer. Insulin was measured using an ADVIA Centaur XP immunoassay system clinical analyzer, and the sensitivity of the assay was 0.2 U/ml. The intra- and interassay variations for insulin were 5 and 9.8 %, respectively.

### Appetite profile

Appetite ratings were recorded on a visual analog scale (VAS) (100 mm) presented on a meter ruler [12]. The scales were anchored with “not at all” at one end and “extremely” at the other end and were combined with questions regarding feelings of hunger and fullness. The VAS was completed seven times throughout the test day and the control day at 0, 5, 10, 15, 20, 25, and 30 min.

### Statistical analysis

Data are presented as the means ± SDs. The test and control results were compared using paired *t* tests. Sample size was calculated with power and sample size program. We are planning a study of a continuous response variable from matched pairs of study subjects. Prior data indicate that the difference in the response of matched pairs is normally distributed with standard deviation 15 pmol/l. If the true difference in the mean response of matched pairs is 15 pmol/l, we will need to study 10 pairs of subjects to be able to reject the null hypothesis that this response difference is zero with probability (power) 80 %. The type I error probability associated with this test of this null hypothesis is 0.05.

All statistical analyses were performed by using SPSS 19.0 (SPSS Inc), with *α* = 0.05. Statistical significance was set at *p* < 0.05 with a two-tailed test.

## Results

Table 1 shows the clinical characteristics of the healthy volunteers.

**Table 1:** Clinical characteristics of healthy volunteers

	Healthy volunteer
Number (male)	12
Age (y)	32.7 ± 9.3
Body mass index (kg/m^2^)	24.5 ± 2.1
Fasting blood glucose (mmol/l)	5.38 ± 0.31
Systolic pressure (mmHg)	124 ± 8
Diastolic pressure (mmHg)	76 ± 9
Heart rate (beats/min)	78 ± 11

**Fig. 1:**
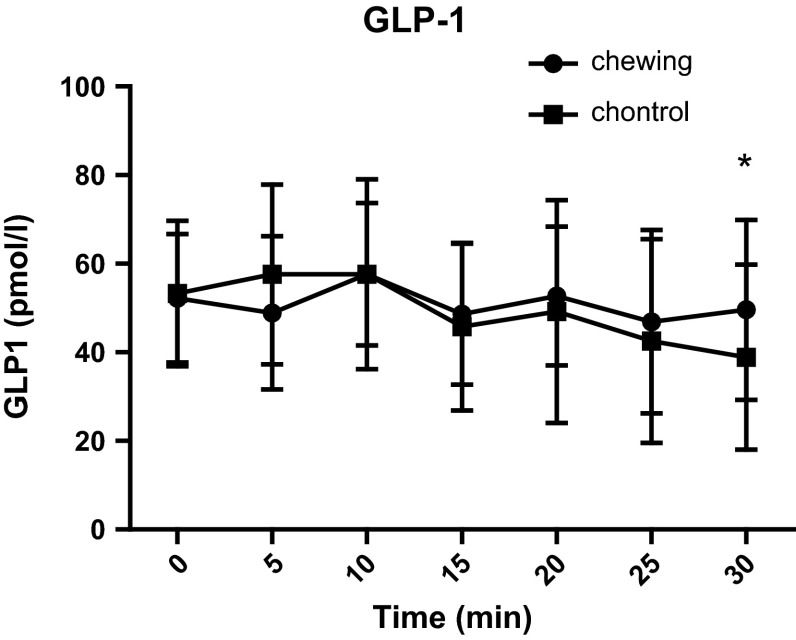
This figure shows the GLP-1 concentration for the chewing group and the control group (*n* = 12). Plasma GLP-1 concentrations 30 min after chewing were significantly increased in the gum-chewing group compared with the non-chewing group (*p < 0.05)

**Fig. 2:**
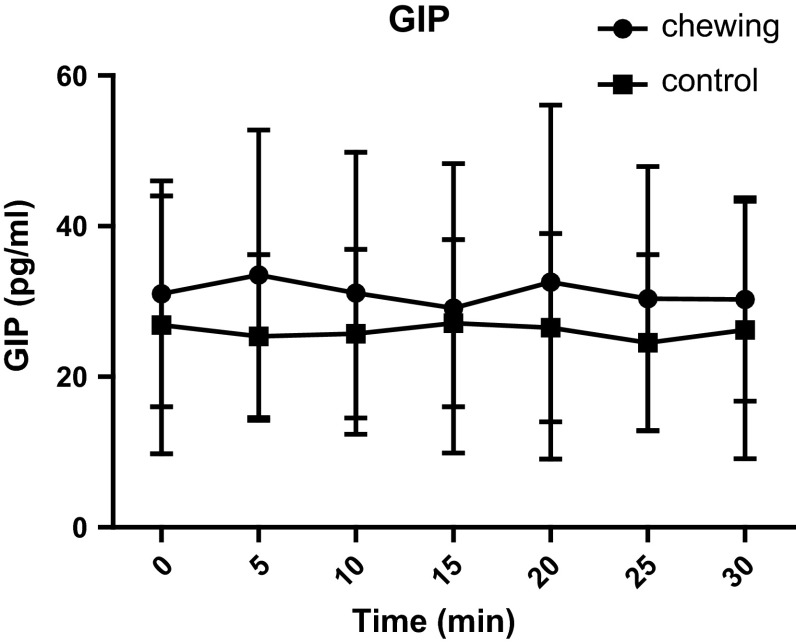
This figure compares the GIP concentration of the chewing group and the control group (*n* = 12). There was no difference between the gum-chewing group and the non-chewing group

Table 2 and Figs. 1, 2 show the comparisons of plasma glucose, serum insulin, plasma active GLP-1, and GIP concentrations between the chewing and non-chewing occasions. In both occasions, plasma glucose, serum insulin, and GIP concentrations were equivalent. Paired t-tests showed no significant differences between the two occasions. Similarly, plasma GLP-1 concentrations 30 min after chewing were significantly increased compared with the non-chewing group (*p < 0.05; 49.6 ± 20.3 pmol/l for the groups that chewed gum vs. 38.9 ± 20.9 pmol/l for the groups that did not chew gum).

**Table 2:** Comparison of glucose, insulin, GLP-1, and GIP between the gum-chewing and non-chewing occasions of healthy volunteers

	0 min	5 min	10 min	15 min	20 min	25 min	30 min
Glucose (mmol/l)							
Chewing	5.4 ± 0.3	5.4 ± 0.6	5.5 ± 0.4	5.6 ± 0.5	5.5 ± 0.5	5.5 ± 0.5	5.6 ± 0.6
Non-chewing	5.3 ± 0.4	5.5 ± 0.5	5.5 ± 0.6	5.5 ± 0.4	5.3 ± 0.6	5.5 ± 0.4	5.5 ± 0.5
Insulin (uIU/ml)							
Chewing	10.1 ± 5.2	9.8 ± 5.7	8.9 ± 5.7	10.4 ± 6.2	9.5 ± 6.0	9.6 ± 5.8	9.1 ± 5.0
Non-chewing	9.4 ± 3.7	8.6 ± 3.5	8.3 ± 4.0	8.8 ± 3.4	8.2 ± 4.6	8.2 ± 4.1	8.1 ± 4.7
GLP-1 (pmol/l)							
Chewing	52.2 ± 14.5	48.9 ± 17.3	57.6 ± 16.1	48.6 ± 15.9	52.7 ± 15.7	46.9 ± 20.7	49.6 ± 20.3*
Non-chewing	53.3 ± 16.4	57.6 ± 20.3	57.6 ± 21.4	45.8 ± 18,9	49.2 ± 25.2	42.5 ± 23.0	38.9 ± 20.9*
GIP (pg/ml)							
Chewing	31.0 ± 15.0	33.5 ± 19.3	31.1 ± 18.7	29.1 ± 19.2	32.6 ± 23.5	30.4 ± 17.5	30.3 ± 13.5
Non-chewing	26.9 ± 17.1	25.4 ± 10.8	25.7 ± 11.2	27.1 ± 11.1	26.5 ± 12.5	24.5 ± 11.7	26.2 ± 17.1

* *p* < 0.05

**Fig. 3:**
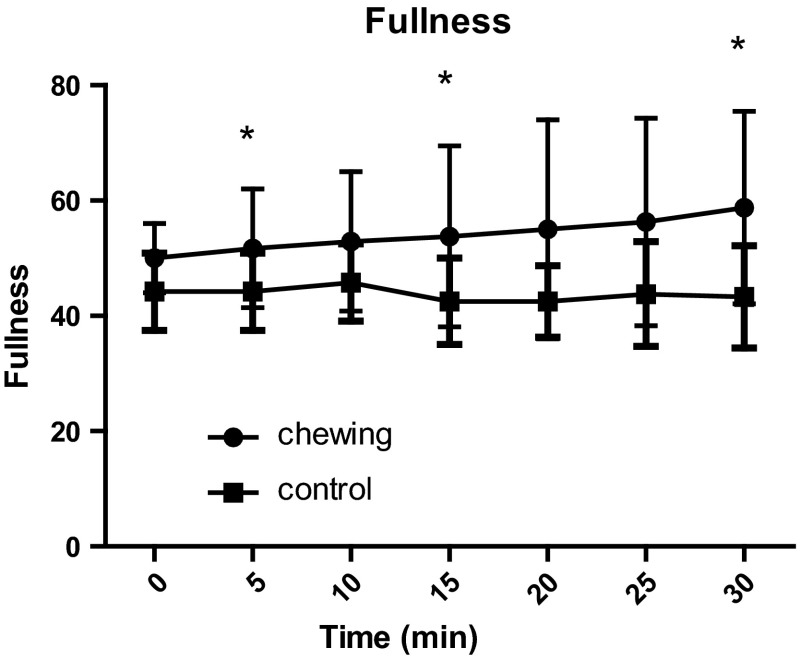
This figure shows the fullness ratings of the chewing group and the control group (*n* = 12). Fullness at 5, 15, and 30 min after the start of the session was significantly increased in the gum-chewing group compared with the non-chewing group (*p < 0.05)

**Fig. 4:**
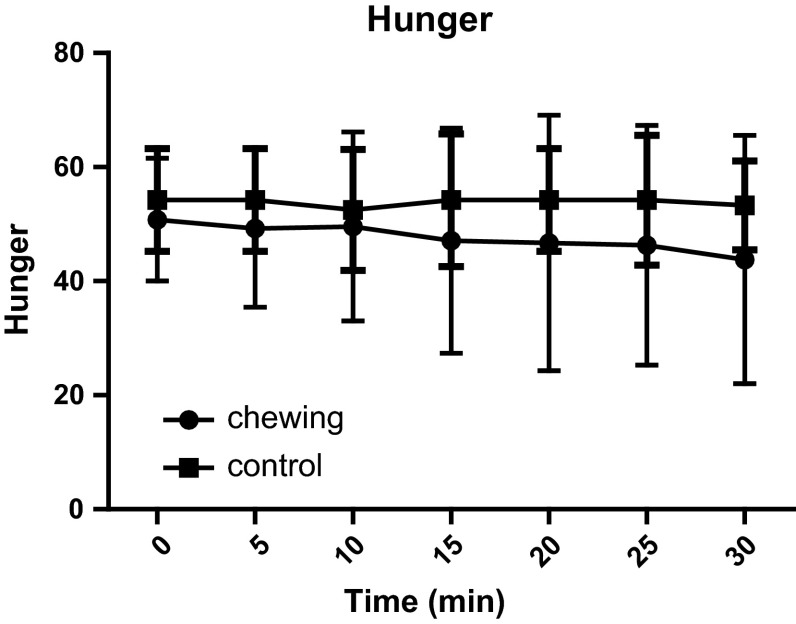
This figure shows the hunger ratings of the chewing group and the control group (*n* = 12). There was no difference between the gum-chewing group and the non-chewing group

Figures 3 and 4 show the comparison of fullness and hunger between the chewing and non-chewing groups. A paired *t* test showed a significant difference in fullness at 5, 15, and 30 min. The chewing group showed a significant increase in fullness compared with the non-chewing group (*p < 0.05; 51.7 ± 10.3, 53.8 ± 15.7, and 58.5 ± 16.7 for the group that chewed gum at 5, 15, and 30 min, respectively, vs. 44.2 ± 6.7, 42.5 ± 7.5, and 43.3 ± 8.9 for the group that did not chew gum at 5, 15, and 30 min, respectively). Hunger ratings did not differ between the chewing and non-chewing groups.

## Discussion

Taste stimuli have a clear stimulating effect on satiety; therefore, gum chewing is considered an effective weight control method because it has the potential to control appetite and food intake.

Gum chewing can provide taste stimuli, and because each piece of gum contains only 5–10 kcal of energy, gum chewing results in a net 11 ± 3 kcal/h increase in energy expenditure [13]. We observed the effect of chewing hard, sugarless gum on the GLP-1, and GIP concentration of healthy volunteers. Our results show that chewing gum 80 times every 2 min during a fasting state made the blood GLP-1 level of the chewing occasion decreasing more slowly than that of the non-chewing group, and at 30 min of chewing, the difference was significant. Furthermore, fullness was increased at 5, 15, and 30 min after chewing compared with non-chewing controls. For volunteers’ blood glucose level and insulin concentration had no different after chewing, we can conclude that change of GLP-1′s concentration is not originated from blood glucose’s change. At the same time, GIP’s concentration has no different after chewing, so change of GLP-1′s concentration is independent of GIP’s level. Our results are consistent with those from the research of Kokkinos et al. [14].

So we speculate that nerve system can regulate GLP-1′s secretion. It has been known for more than 20 years that GLP-1 can be synthesized in the mammalian brain [15]. Some studies demonstrated that PPG neurons are non-adrenergic neurons with their cell bodies located exclusively in the caudal nucleus of the solitary tract (NTS), the caudal medullary reticular formation and the olfactory bulb [16, 17]. These studies also demonstrated a widespread projection pattern for these neurons, with the highest density of terminals observed in the paraventricular nucleus (PVN) and the dorsomedial hypothalamus (DMH) [16, 18]. At present, the nature of the link between the GLP-1 of the central nervous system and the postprandial release of peripheral GLP-1 and whether intestinal GLP-1 can enter the brain to fully activate the GLP-1 receptor remain controversial. Our research suggests that chewing can stimulate central nervous system and effect GLP-1′s level without food impacting. If GLP-1 was secreted from central nervous system itself is not know. But according past study, it has a possibility that the increased GLP-1 comes from central nervous system.

The literature relating chewing gum to energy intake is limited and nuanced by methodological variations. Studies have examined the influence of gum chewing on body weight, but the results are not consistent. Different methods of chewing gum may lead to different effects. No effects have been observed when chewing was set at a fixed time (2 h after a meal) or in response to hunger [19]. Mixed findings have been reported from chewing gum immediately prior to a meal [19, 20]. Chewing gum may not decrease food intake in all people. In addition, chewing sweet gum can increase hunger [21] because it stimulates saliva secretion; thus, chewing gum can stimulate rather than inhibit eating [22]. In 2012, scholars in the United States make 102 overweight or obese adult volunteers to chew gum 90 min per day for 8 weeks, and the result shows that this did not facilitate weight loss in these overweight and obese adults [23].

In Japan, the practice of thorough mastication (for example, 30 chews per bite) has been shown to be an effective behavioral approach for curbing obesity [24] because the mastication-induced activation of histamine neurons suppresses physical food intake through the H1-receptor in the hypothalamic paraventricular nucleus and the ventromedial hypothalamus, which are known as satiety centers [25]. By chewing slowly, healthy women can reduce calorie intake [26].

The present study shows that chewing gum induced changes in GLP-1 concentration independently of changes in blood glucose levels. At the same time, the GIP level did not change with the change in GLP-1, which suggests that the chewing action itself may be stimulate the secretion of GLP-1; the nervous system regulates GLP-1 secretion independently of changes in GIP, and the action of chewing has no effect on the secretion of GIP.

The effect of gum chewing on satiety emerges earlier than the changes in blood hormones. After chewing for 5, 15, and 30 min, the chewing group’s satiety was significantly higher than that of the control group. The blood GLP-1 level on experiment days was always higher than that on control days, and it was significantly higher in the experimental group than the control group after chewing for 30 min. There was no significant difference in GLP-1 concentration in the test group at each time point, a result that was consistent with the volunteers’ self-reports. Hunger levels did not differ significantly between the two groups; further research can measure the plasma ghrelin concentrations to verify this finding. In this experiment, the blood sample quantity was limited, and we did not measure other gastrointestinal hormones. Research shows that chewing sugarless gum can increase satiety; therefore, gum chewing may be a useful way to lose weight.

Some scholars suggest that the effects of neuropeptide GLP-1 (released by PPG neurons) are distinct from the effects of incretin GLP-1 (released by enteroendocrine cells) and that the PPG neurons constitute a central signaling network that integrates peripheral and central signals for both long- and short-term nutritional and digestive status. GLP-1 neurons might produce an output signal to feeding and autonomic circuits that optimizes digestion and the assimilation of nutrients and regulates calorific intake [27]. We speculate that the chewing action itself may stimulate the central PPG neurons to promote the release of GLP-1.

## Conclusion

Among healthy men in a fasting state, chewing sugarless gum can increase satiety with no effect on blood glucose and can decrease the decline of GLP-1 concentration. Chewing gum has no significant effect on blood insulin and GIP concentration. The present study suggests that chewing sugarless gum may be an economical and effective method to help obesity patients control their energy intake and decrease weight with no changes in calorie intake. Although there are different opinions about this benefit of gum chewing, our study showed positive results, and it is worth conducting a large-scale clinical research study to verify the effectiveness of this method.
